# Factors associated with tobacco use among Saudi Arabian youth: Application of the Theory of Planned Behavior

**DOI:** 10.18332/tid/196678

**Published:** 2024-12-18

**Authors:** Mosa A. Shubayr, Anwar S. Alhazmi, Mervat M. El Dalatony, Eman D. El Desouky, Abdulmohsen H. Al-Zalabani, Sarah S. Monshi, Ahmed A. Elkhobby, Shaikha K. Aldukhail, Mohammed M. Alqahtani, Mohammed S. Aldossary

**Affiliations:** 1 Department of Preventive Dental Sciences, College of Dentistry, Jazan University, Jazan, Saudi Arabia; 2 Public Health and Community Medicine Department, Faculty of Medicine, Menoufia University, Shibin El Kom, Egypt; 3 General Directorate of Research and Studies, Ministry of Health, Riyadh, Saudi Arabia; 4 Department of Family and Community Medicine, College of Medicine, Taibah University, Medina, Saudi Arabia; 5 Department of Health Administration and Hospitals, College of Public Health and Health Informatics, Umm Al-Qura University, Mecca, Saudi Arabia; 6 Tobacco Control Program, Ministry of Health, Riyadh, Saudi Arabia; 7 Department of Preventive Dental Sciences, College of Dentistry, Princess Nourah bint Abdulrahman University, Riyadh, Saudi Arabia; 8 Department of Respiratory Therapy, College of Applied Medical Sciences, King Saud bin Abdulaziz University for Health Sciences, Riyadh, Saudi Arabia

**Keywords:** tobacco use, global youth tobacco survey, Saudi Arabian youth, Theory of Planned Behavior

## Abstract

**INTRODUCTION:**

The epidemic of tobacco use poses one of the most severe public health challenges globally, responsible for over 8 million deaths annually according to the World Health Organization (WHO). This study investigates the factors associated with tobacco use among Saudi Arabian youth using the Theory of Planned Behavior (TPB) framework.

**METHODS:**

A cross-sectional study used the 2022 Global Youth Tobacco Survey (GYTS) to assess tobacco use among 5610 Saudi students aged 13–15 years. The structured, anonymous questionnaire collected demographic data and TPB constructs (e.g. attitudes, subjective norms, perceived behavioral control, and intentions) were derived from the validated GYTS questionnaire, with items grouped and scored to represent each construct. One-way ANOVA, t-tests, and logistic regression analyses were conducted, with a significance level set at 0.05.

**RESULTS:**

Of the surveyed students, 5610 (80.4%) aged 13–15 years met the inclusion criteria. Tobacco use was prevalent, with 31.3% having ever used tobacco and 8.78% currently using it. Negative attitudes toward tobacco were inversely associated with usage (AOR=0.97; 95% CI: 0.970–0.974). Subjective norms, reflecting perceived social pressure, were positively associated with tobacco use (AOR=1.17; 95% CI: 1.170–1.176). Perceived behavioral control, indicating the ease of quitting or avoiding tobacco, was strongly associated with use (AOR=1.87; 95% CI: 1.848–1.888). Stronger behavioral intentions to use tobacco were also associated with a higher likelihood of use (AOR=1.24; 95% CI: 1.226–1.246).

**CONCLUSIONS:**

The high prevalence of tobacco use among Saudi Arabian youth, early initiation, and significant social influences highlight the need for comprehensive public health strategies. Targeted education, reduced tobacco accessibility, and programs to shift social norms and attitudes are essential. Addressing these factors can help prevent initiation and support cessation efforts among young people.

## INTRODUCTION

The epidemic of tobacco use is considered one of the world’s most severe public health challenges, accounting for over 8 million deaths annually, as reported by the World Health Organization (WHO). This overwhelming death toll includes more than 7 million smokers and approximately 1.3 million individuals exposed to secondhand smoke, underlining the lethal impact of tobacco consumption^1^. The majority, about 80%, of the world’s 1.3 billion tobacco users, reside in low- and middle-income countries, where the burden of tobacco-related health issues is most pronounced. In 2020, tobacco was used by 22.3% of the global population with a significant gender disparity: 36.7% of men compared to 7.8% of women. To combat this escalating crisis, WHO member states ratified the WHO Framework Convention on Tobacco Control (WHO FCTC) in 2003, an agreement currently supported by 182 countries. The WHO MPOWER measures, which are defined as monitoring tobacco use (M), preventing tobacco exposure (P), offering help to quit (O), warning (W), enforcing (E), and raising taxes (R), align with the WHO FCTC and have demonstrated effectiveness in saving lives and reducing healthcare costs by averting tobacco-related expenditures^1^.

Tobacco consumption among young people poses a critical challenge for public health systems worldwide as it threatens to reverse decades of progress against preventable diseases and mortality. The worldwide trend of tobacco consumption presents a sharp contrast; although some countries have seen notable reductions in smoking rates due to robust public health initiatives and legislative actions, others, including developing countries and some high-income nations such as Saudi Arabia, have witnessed alarming increases^2^. This situation highlighted an urgent need for targeted tobacco control measures, particularly in countries like Saudi Arabia, where smoking rates have been on the rise^2^.

In Saudi Arabia, the prevalence of tobacco use presents a complex public health dilemma, reflecting the global crisis yet intensified by specific sociocultural and regulatory contexts; approximately 9.72% to 37% among secondary school students, 2.4% to 30.9% among college students, and 12.7% to 39.6% among the adolescent age group, regardless of educational stage^2^. Recent data from the WHO revealed that in 2016, 21.2% of male adolescents and 9.1% of female adolescents in Saudi Arabia were smokers, highlighting a concerning trend that necessitates urgent intervention^3^. Predictions based on current trends suggest a notable increase in smoking rates among Saudi youths by 2025, emphasizing the need for effective tobacco control strategies^3^. The early initiation of smoking during adolescence, coupled with the challenges in cessation, highlights the need to understand and address the multifaceted factors influencing youth tobacco use, such as family, social, and psychological factors^4-6^. Despite significant efforts by Saudi Arabia to combat tobacco smoking, the persistent high prevalence of tobacco use calls for immediate action from both policymakers and health professionals^7-10^.

The context of this research is further explained by Saudi Arabia’s Vision 2030, which prioritizes public health and preventive care^11^. Saudi Arabia’s adherence to the WHO FCTC and implementation of MPOWER strategies reflect a proactive stance against the tobacco epidemic. Nonetheless, the surge in smoking rates among Saudi youth reveals a critical gap in the understanding of this demographic’s tobacco use drivers, underlining the need for research that informs more effective public health strategies^12^.

The Theory of Planned Behavior (TPB) is a theory-based structure^13^ that emphasizes attitude toward the behavior, subjective norm, and perceived behavioral control to predict human behavior and behavioral intention. It consists of six main constructs: behavioral belief, behavioral outcomes, normative belief, motivation to comply, control beliefs, and perceived power^13,14^.

This study addresses a critical public health challenge in Saudi Arabia – tobacco use among youth. By understanding the behavioral drivers of smoking, the findings of this study can inform evidence-based interventions and policy reforms that align with the goals of Vision 2030. Addressing youth tobacco use is an important step in advancing Saudi Arabia’s commitment to healthcare prevention and health promotion^12^. Accordingly, this study adopts the TPB framework to examine the factors linked to tobacco use among Saudi Arabian youth.

## METHODS

### Study design and sample

This study was conducted using the cross-sectional Global Youth Tobacco Survey (GYTS) to assess tobacco usage prevalence among the youth population in Saudi Arabia. The survey, carried out in 2022 by the Ministry of Health and the Ministry of Education, achieved a high response rate of 92.3%. Of the respondents, 6983 were students from grades 1–3 of intermediate education, and were eligible for the study. Among these, data from 5610 students, specifically those aged 13–15 years, are analyzed and reported, focusing on this age group’s tobacco use patterns.

### Data collection process

The data collection for this study was systematically conducted using the structured and standardized questionnaire provided by the Global Youth Tobacco Survey (GYTS). The process began with the selection of schools across Saudi Arabia through a two-stage cluster sampling method, ensuring a representative sample of the student population. Within each selected school, equal probability sampling (with a random start) of classes was conducted from each school that participated in the survey. All classes in the selected schools were included in the sampling frame. All students in the selected classes from grades 1–3 of intermediate level were invited to participate in the survey.

The GYTS questionnaire, designed to capture a comprehensive range of information on tobacco use among youths, was distributed to the students in a paper-based format. To ensure the anonymity and confidentiality of respondents’ answers, the surveys were self-administered by the students using scannable answer sheets that did not require any personal identification information.

Data collection was supervised by trained personnel from the Ministry of Health and the Ministry of Education, who also provided instructions to the students on how to accurately complete the questionnaire.

Following the completion of the surveys, the answer sheets were collected and securely transported for data processing. The overall response rate was calculated based on the number of students who provided complete questionnaires out of the total eligible participants. The data collection commenced after the ethics approval was obtained from the Central Institutional Review Board of the Saudi Ministry of Health (MOH) (IRB: 23-106 M).

### Study instrument

The questionnaire from the Global Youth Tobacco Survey (GYTS) was developed after a thorough review of pertinent literature on tobacco use and the control guidelines set by the WHO. This questionnaire included sections on tobacco use (smoking and smokeless products), cessation, secondhand smoke (SHS), anti-tobacco advertising and promotion, access to and availability of tobacco products, and knowledge and attitudes regarding tobacco use. It captured demographic details, and the six constructs of the Theory of Planned Behavior (TPB) related to tobacco use.

### Study variables and measures

*Main outcome*

The main outcome was current tobacco users including smoked and smokeless tobacco.

*Demographic characteristics*

Three items were included in the questionnaire to elicit personal information on academic grade, sex and age.

*TPB constructs*

The TPB constructs were all derived from GYTS and aimed to explore tobacco use among youths with a comprehensive set of 26 items distributed across: intention (3 items), attitudes (7 items), subjective norms (9 items), perceived behavioral control (PBC) (2 items), alongside awareness and knowledge (16 items) (Supplementary file).

Awareness and knowledge

This section assessed knowledge about health hazards of smoking harmful effects, awareness of messages that are against using tobacco and awareness of advertisements or promotions for tobacco use, where scores could reach up to 16, indicating stronger awareness and knowledge on tobacco use.

Intention

This was through items asking about future tobacco use, aiming to estimate the likelihood of engaging in such behavior within the next year. A 4-point scale from ‘unlikely’ to ‘likely’ helped in quantifying responses, where scores could reach up to 12, indicating stronger intentions towards tobacco use.

Attitudes

Attitudes towards tobacco use were examined through 7 items reflecting behavioral beliefs and outcome evaluations, such as the perceived enjoyment and difficulty of quitting. Responses were measured on a scale ranging from ‘unlikely’ to ‘likely’ for behavioral beliefs. A total score of 27 could be obtained indicating a non-favorable attitude towards use of tobacco (higher scores indicates high likelihood of smoking and difficult quitting).

Subjective norms

These were evaluated through 9 items about the perceived expectations of significant others regarding the respondent’s tobacco use, including both normative beliefs and motivation to comply. A maximum score of 33 was possible, with higher scores indicating stronger perceived pressure from important others.

Perceived behavioral control (PBC)

PBC over tobacco use was assessed with 2 items addressing control beliefs and perceived power, such as confidence in one’s ability to abstain from tobacco use. This section also employed a 4-point scale, with scores up to 8 indicating higher perceived control.

### Internal consistency and reliability of the questionnaire items

To validate the proposed model, the internal consistency and reliability of the questionnaire items were assessed using Cronbach’s alpha. Confirmatory factor analysis (CFA) was conducted to validate the underlying factor structure of the questionnaire.

Cronbach’s alpha ranges from 0 to 1, with higher values indicating greater internal consistency. Cutoff values for interpreting Cronbach’s alpha were based on the guidelines provided by George and Mallery^15^: ≥0.9 is considered ‘excellent’, 0.8–0.9 is ‘good’, 0.7–0.8 is ‘acceptable’, 0.6–0.7 is ‘questionable’, 0.5–0.6 is ‘poor’, and <0.5 is ‘unacceptable’. However, for exploratory research, a Cronbach’s alpha value of ≥0.5 is considered acceptable; Nunnally^16^ suggested that in early research, reliability coefficients of 0.5–0.6 would be sufficient. Therefore, in the context of this exploratory study, Cronbach’s alpha values of ≥0.5 were considered acceptable. In our research Cronbach’s alpha for all items were within the acceptable limits (0.6–0.8).

### Construct validity

Principal component analysis (PCA) was done initially to explore the underlying factor structure and assess the dimensionality of the questionnaire (Supplementary file), followed by confirmatory factor analysis (CFA) was conducted to test the construct validity of the model. The CFA was performed using Amos 26 software. In our research, loadings were within the acceptable range (0.602–0.941), with (AVE) average variance extracted (AVE) values >0.5, acceptable according to Fornell and Larcker^17^.

### Data analyses

The survey responses were pre-coded based on a predetermined coding sheet for efficient data entry into the database. The data were prepared by the Centre for Disease Control and Prevention (CDC, Atlanta, US). Descriptive statistics used absolute numbers and weighted percentages for categorical data.

To validate the questionnaire based on the TPB model, two analysis methods were employed: confirmatory factor analysis (CFA) and principal component analysis (PCA). CFA was used to assess the factor structure and measurement validity of the TPB constructs. PCA was conducted to explore the underlying factor structure and assess the dimensionality of the questionnaire. PCA with varimax rotation was applied. The number of factors retained was determined based on eigenvalues >1 and examination of the scree plot. Factor loadings, communalities, and explained variances were examined to assess the dimensionality of the questionnaire. Factor loadings were examined, and reliability of the constructs was assessed using Cronbach’s alpha coefficients. Scores of constructs are presented as mean and standard deviation (SD). To explore the associations of different factors with TPB construct scores, one-way ANOVA and t-tests were conducted. Logistic regression analyses, adjusted for age, gender, grade level, average spending, awareness and knowledge, attitude, subjective norms, PBC, and behavioral intentions were used to assess associations with tobacco use. A significance level of 0.05 was set for all tests. All statistical analyses were performed using IBM SPSS Advanced Statistics (Statistical Package for Social Sciences), version 27 (Armonk, NY: IBM Corp), and IBM SPSS *Amos*™ (Chicago: IBM SPSS.) software.

## RESULTS

A total of 6983 intermediate students from grades 1–3 completed the survey. Among them, 5610 students (80.4%) aged 13–15 years from 95 selected schools and 275 sampled classes actively participated in the survey. Gender distribution was fairly balanced (52.4% male, 47.6% female). The largest age group was 14 years (38.1%), followed by those aged 13 years (34.4%) and those aged 15 years (27.5%). Most participants were in the second intermediate grade (40.2%), with the first and third intermediate grades nearly equally represented at 30.0% and 29.8%, respectively. In terms of weekly spending, 57.3% of the participants reported spending no money or <30 SAR, while 42.7% spent ≥30 SAR. Specifically, 22.7% usually did not have any spending money, 34.6% spent <30 SAR, 20.2% spent 30–49 SAR, 13.5% spent 50–99 SAR, and 9.1% spent ≥100 SAR per week (Table 1).

**Table 1:** Demographic characteristics of participants (N=5610)

*Characteristics*	n^a^ (%)*
**Gender**	
Male	2941 (52.4)
Female	2669 (47.6)
**Age** (years)	
13	1928 (34.4)
14	2140 (38.1)
15	1542 (27.5)
**Grade**	
1st intermediate	1684 (30.0)
2nd intermediate	2253 (40.2)
3rd intermediate	1673 (29.8)
**Average spending per week** (SAR)	
No spending or <30	3200 (57.3)
≥30	2360 (42.7)
**Detailed spending categories** (SAR)	
I usually don’t have any spending money	1266 (22.7)
<30	1934 (34.6)
30–49	1128 (20.2)
50–99	741 (13.5)
≥100	491 (9.1)

SAR: 1000 Saudi Arabian Riyals about US$270.

* There are missing data and percentages are represented as a valid percent.

a Unweighted number of participants.

Table 2 highlights the significant prevalence of tobacco use among Saudi Arabian youth. About 31.3% of participants had ever used tobacco, with 8.78% being current users. The use of tobacco products other than cigarettes (including shisha, heated, and smokeless), was also common, with 8.49% currently using such products. Among various forms of tobacco, smoked tobacco was predominant, with 6.75% currently smoking and 23.99% having ever smoked. shisha smoking was noted, with 2.80% currently smoking shisha and 14.77% having ever smoked it. Heated tobacco products and smokeless tobacco were less commonly used, with current usage rates of 1.56% and 3.33%, respectively. However, a significant number of participants had ever used these products, at 7.62% for heated tobacco and 8.93% for smokeless tobacco.

**Table 2:** Tobacco use among Saudi Arabian youth (N=5610)

*Characteristics*	*n^a^ (%)*
**Any tobacco used** (smoked, heated, and/or smokeless)	
Current users	495 (8.78)
Ever users	1767 (31.33)
Current users of other products other than cigarettes	479 (8.49)
**Smoked tobacco**	
Current tobacco smokers	381 (6.75)
Current cigarette smokers	159 (2.82)
Frequent cigarette smokers	23 (0.41)
Current smokers of other tobacco	293 (5.20)
Currently smoke shisha	158 (2.80)
Ever tobacco smokers	1353 (23.99)
Ever cigarette smokers	727 (12.89)
Ever smokers of other tobacco	1064 (18.86)
Ever smokers of shisha	833 (14.77)
**Heated tobacco products**	
Current users	88 (1.56)
Ever users	430 (7.62)
**Smokeless tobacco**	
Current users	188 (3.33)
Ever users	504 (8.93)
**Susceptibility**	
Susceptible to using tobacco in the future	340 (6.03)
Never tobacco smokers who thought they might enjoy smoking	848 (15.04)
**Age tried shisha smoking** (years)	
≤7	89 (1.58)
8–9	63 (1.12)
10–11	76 (1.35)
12–13	155 (2.75)
14–15	145 (2.57)
**Age tried using heated tobacco products** (years)	
≤7	28 (0.50)
8–9	14 (0.25)
10–11	16 (0.28)
12–13	16 (0.28)
14–15	15 (0.27)
**Age tried smokeless tobacco** (years)	
≤7	56 (0.99)
8–9	21 (0.37)
10–11	17 (0.30)
12–13	24 (0.43)
14–15	21 (0.37)
**Cigarettes per day**	
<1	57 (1.01)
1	42 (0.74)
2–5	39 (0.69)
6–10	9 (0.16)
11–20	4 (0.07)
>20	7 (0.12)
**Age ever tried cigarettes** (years)	
≤7	85 (1.51)
8–9	96 (1.70)
10–11	91 (1.61)
12–13	133 (2.36)
14–15	91 (1.61)

a Unweighted number of participants.

About 6.03% of participants were susceptible to future tobacco use, and 15.04% of non-smokers thought they might enjoy smoking. The initiation age for shisha and other tobacco products started as young as ≤7 years. Notably, 1.58% started shisha smoking at ≤7 years, with the highest initiation at 12–13 years (2.75%). Similarly, early initiation was with heated tobacco (0.50% by 7 years) and smokeless tobacco (0.99% by 7 years). Most cigarette users consumed less than one cigarette per day (1.01%). The initiation age for cigarettes was also alarming, with 1.51% trying cigarettes at ≤7 years, increasing to 2.36% at the ages of 12–13 years.

Table 3 presents the distribution of scores for the TPB constructs of intention, attitude, subjective norm, PBC, and awareness and knowledge, as factors associated with tobacco use among Saudi Arabian youth. The reliability of the measures, indicated by Cronbach’s alpha, showed moderate to high reliability across constructs. The intention to avoid tobacco use had a mean score of 3.4 (SD=1.1) on a scale 3–12, with a Cronbach’s alpha of 0.73. Attitudes towards tobacco use had a mean score of 14.2 (SD=4.3) on a scale 7–27, with a Cronbach’s alpha of 0.70. Subjective norms had a mean score of 7.3 (SD=2.7) on a scale 9–33, with a Cronbach’s alpha of 0.80. PBC had a mean score of 2.2 (SD=0.7) on a scale 2–8, with a Cronbach’s alpha of 0.60. Awareness and knowledge about the health effects and risks of tobacco use had a mean score of 6.5 (SD=2.3) out of a maximum of 16, with a Cronbach’s alpha of 0.60.

**Table 3:** Scores of TPB constructs as factors associated with tobacco use among Saudi Arabian youth (N=5610)

*Construct*	*Final* *number* *of items*	*Mean* *(SD)*	*Range*	*Cronbach* *α*
Awareness and knowledge	16	6.5 (2.3)	0–16	0.60
Attitudes	7	14.2 (4.3)	7–27	0.70
Subjective norms	9	7.3 (2.7)	9–33	0.80
PBC	2	2.2 (0.7)	2–8	0.60
Behavioral intentions	3	3.4 (1.1)	3–12	0.73

TPB: Theory of Planned Behavior. PBC: perceived behavior control.

Table 4 details the associations of the TPB constructs among Saudi Arabian youth, showing scores for awareness and knowledge, attitude, subjective norms, PBC, and behavioral intentions across different age groups, genders, and smoking statuses. Scores generally increased with age, with those aged 15 years having higher mean scores in all constructs. Males had a mean awareness and knowledge score of 6.6 (SD=2.4) and an attitude score of 13.7 (SD=4.1), while females had a mean awareness and knowledge score of 6.5 (SD=2.3) and a higher attitude score of 14.8 (SD=4.3). Both genders had similar subjective norms, with males slightly higher in perceived control (2.3 vs 2.2).

**Table 4:** Associations between the TPB constructs and the general personal characteristics of the Saudi Arabian youth (N=5610)

*Variables*	*Awareness and knowledge*	*Attitude*	*Subjective norms*	*PBC*	*Behavioral intentions*
	*Mean ± SD*	*Mean ± SD*	*Mean ± SD*	*Mean ± SD*	*Mean ± SD*
**Age**^a^ (years)					
13	6.4 ± 2.3	14.1 ± 4.2	7.1 ± 2.4	2.2 ± 0.6	3.3 ± 0.9
14	6.5 ± 2.3	14.3 ± 4.3	7.3 ± 2.7	2.2 ± 0.7	3.4 ± 1.1
15	6.8 ± 2.3	14.3 ± 4.3	7.5 ± 3.0	2.3 ± 0.9	3.5 ± 1.3
**Gender**					
Male	6.6 ± 2.4	13.7 ± 4.1	7.3 ± 2.7	2.3 ± 0.8	3.4 ± 1.2
Female	6.5 ± 2.3	14.8 ± 4.3	7.3 ± 2.7	2.2 ± 0.7	3.3 ± 1.1
**Current tobacco smokers**					
No	6.5 ± 2.3	14.1 ± 4.2	7.0 ± 2.1	2.1 ± 0.5	3.2 ± 0.8
Yes	7.0 ± 2.5	15.4 ± 4.2	11.4 ± 5.4	3.5 ± 1.6	5.3 ± 2.5
**Current cigarette smokers**					
No	6.5 ± 2.3	14.2 ± 4.2	7.1 ± 2.3	2.2 ± 0.6	3.3 ± 0.9
Yes	6.8 ± 2.6	15.9 ± 4.2	13.4 ± 6.0	4.4 ± 1.7	6.6 ± 2.6
**Ever tobacco smokers**					
No	6.4 ± 2.3	14.0 ± 4.3	6.7 ± 1.7	2.1 ± 0.3	3.1 ± 0.4
Yes	6.9 ± 2.4	14.9 ± 4.1	9.0 ± 4.2	2.7 ± 1.2	4.2 ± 1.9
**Ever cigarette smokers**					
No	6.5 ± 2.3	14.1 ± 4.3	6.9 ± 2.0	2.1 ± 0.4	3.1 ± 0.6
Yes	6.9 ± 2.5	14.8 ± 4.2	9.9 ± 4.8	3.1 ± 1.5	4.8 ± 2.2

TPB: Theory of Planned Behavior. PBC: perceived behavior control.

a ANOVA test, all other comparisons done by t-test. All p-values for comparisons were significant at <0.001.

Current tobacco smokers scored higher than non-smokers in all constructs, with a mean awareness and knowledge score of 7.0 (SD=2.5), an attitude score of 15.4 (SD=4.2), subjective norms score of 11.4 (SD=5.4), a PBC score of 3.5 (SD=1.6), and behavioral intentions score of 5.3 (SD=2.5). Current cigarette smokers had higher scores in PBC (4.4 vs 2.2). Ever smokers had higher scores than never smokers in all constructs, with a mean awareness and knowledge score of 6.9 (SD=2.4), an attitude score of 14.9 (SD=4.1), subjective norms score of 9.0 (SD=4.2), a PBC score of 2.7 (SD=1.2), and behavioral intentions score of 4.2 (SD=1.9). Ever cigarette smokers scored higher in all constructs, with a mean PBC score of 4.8 (SD=2.2). All p-values for comparisons were significant at <0.001, indicating strong associations between these TPB constructs and Saudi Arabian youth’s demographic and behavioral factors.

Table 5 presents the logistic regression analysis results identifying factors that predict current tobacco use among Saudi Arabian youth. Age was significantly associated with tobacco use, with 14-year-olds being less likely to use tobacco compared with 13-year-olds (AOR=0.87; 95% CI: 0.85–0.89, p<0.001). Males were more likely than females to use tobacco (AOR=1.26; 95% CI: 1.24–1.28, p<0.001). Grade level also played a role, with students in the second intermediate grade (AOR=1.08; 95% CI: 1.05–1.11, p<0.001) and third intermediate grade (AOR=1.17; 95% CI: 1.13–1.21, p<0.001) showing higher odds of tobacco use compared with those in the first intermediate grade. Higher average spending was linked to increased tobacco use (AOR=1.12; 95% CI: 1.10–1.14, p<0.001).

**Table 5:** Factors predicting current use of tobacco products among Saudi Arabian youth (N=5610)

*Variables*	*AOR (95% CI)*	*p*
**Age** (years)		
13 ®	1	
14	0.87 (0.85–0.89)	<0.001
15	0.98 (0.95–1.01)	0.163
**Gender**		
Female ®	1	
Male	1.26 (1.24–1.28)	<0.001
**Grade**		
1st intermediate ®	1	
2nd intermediate	1.08 (1.05–1.11)	<0.001
3rd intermediate	1.17 (1.13–1.21)	<0.001
**Average spending**	1.12 (1.10–1.14)	<0.001
**TPB constructs**		
Awareness and knowledge	1.04 (1.04–1.05)	<0.001
Attitude	0.97 (0.97–0.97)	<0.001
Subjective norms	1.17 (1.17–1.18)	<0.001
PBC	1.87 (1.85–1.89)	<0.001
Behavioral intentions	1.24 (1.23–1.25)	<0.001

AOR: adjusted odds ratio; adjusted for age, gender, grade level, average spending, awareness and knowledge, attitudes, subjective norms, PBC, and behavioral intentions. A p<0.05 is considered significant. TPB: Theory of Planned Behavior. PBC: perceived behavior control. ® Reference categories.

Awareness and knowledge about tobacco predicted usage, with higher awareness and knowledge scores linked to greater use (AOR=1.04; 95% CI: 1.04–1.05, p<0.001), while negative attitudes toward tobacco inversely predicted usage (AOR=0.97; 95% CI: 0.97–0.97, p<0.001). Subjective norms, which reflect perceived social pressure, were positively associated with tobacco use (AOR=1.17; 95% CI: 1.17–1.18, p<0.001). PBC, indicating the ease of quitting or avoiding tobacco, was strongly associated with tobacco use (AOR=1.87; 95% CI: 1.85–1.89, p<0.001). Stronger behavioral intentions to use tobacco were also associated with a higher likelihood of use (AOR=1.24; 95% CI: 1.23–1.25, p<0.001).

## DISCUSSION

The findings highlight several factors associated with tobacco use among Saudi Arabian youth via the TPB framework. The prevalence of tobacco use among intermediate school students was alarmingly high, with approximately one-third of the participants having used tobacco and nearly 9% being current users. This is consistent with a 2021 study that found that 80.2% of high school students and 74.6% of middle school students who used tobacco products in the past 30 days reported using a flavored tobacco product during that period^18^. Additionally, this result confirmed a study that found a high proportion of smokers among adolescents in Saudi Arabia^2^. This high prevalence emphasizes the urgency for public health interventions.

The demographic analysis indicated that males were more likely than females to use tobacco, which is consistent with cultural norms in Saudi Arabia, where smoking is more socially acceptable among males^19^. However, the increasing tobacco use prevalence among females signals a concerning trend that must be addressed to prevent a similar epidemic in this group^20^. This rising susceptibility among females may be influenced by changing social dynamics, such as seeing peers or family members smoking as well as being targeted by marketing strategies^20^.

In addition, age and grade level were significantly associated with tobacco use, with older students and those in higher grades being more likely to use tobacco. This mirrors the findings of a study by Al-Otaibi et al.^21^. The increased peer pressure and social influence as students progress through school could explain this trend. The study found that students who felt more social pressure to use tobacco, were more likely to be current users. Additionally, the study by Leshargie et al.^22^ observed that students who experienced peer pressure from their friends were more likely to smoke cigarettes. Initiation rates could be effectively reduced by interventions targeting these age groups and focusing on peer influence. Programs that improve peer support and resilience against peer pressure have shown promise in other contexts and could be adapted for use in Saudi Arabia.

Weekly spending was also linked to tobacco use, with higher spending correlating with increased use. This increase in expenditure might affect not only smokers but also their families^23,24^ as well as healthcare spending^25^, suggesting a role played by economic factors in tobacco accessibility and consumption among the youth. To help mitigate this issue, strategies must be devised to reduce students’ spending or increase the price of tobacco products^26^. Studies have shown that increasing the prices of tobacco products is one of the most effective measures for reducing smoking among youths^27^. Despite the Saudi government’s implementation of a 73.8% increase in overall tobacco taxes, the taxation rates imposed on tobacco products remain below the threshold recommended by the World Health Organization (WHO), which advocates for rates of ≥75% ^28^.

Interestingly, higher awareness and knowledge about the risks of tobacco use was associated with greater prevalence of use. This counterintuitive finding suggests that awareness and knowledge alone is insufficient in deterring tobacco use. This is consistent with a study that found that although medical teachers were generally well-informed about smoking-related knowledge, they may still underestimate the difficulty of quitting smoking^29^. This indicates that more studies should be conducted to explain why people continue smoking despite knowing the risks. In this study, despite students’ awareness of risks, other factors, such as social influence and perceived control, play more significant roles in their decision to use tobacco. This necessitates comprehensive education programs addressing these psychosocial factors^30^. This finding suggests that tobacco control campaigns should not only provide information but also actively engage with youth to address the social and behavioral aspects of tobacco use.

Attitudes toward tobacco use were identified as a significant predictor of its use. Students who held more favorable attitudes toward tobacco were more likely to engage in smoking behaviors. This finding aligns with the results of Tapera et al.^31^, which highlighted the positive association between pro-tobacco attitudes and usage. However, our findings contrast with those of Riyadi et al.^32^ who reported that participants with unfavorable attitudes toward smoking were more likely to smoke compared to those with favorable attitudes. This highlighted the need for interventions that change perceptions and attitudes toward tobacco, making it less desirable and socially acceptable among youth. In addition, campaigns that reshape the image of tobacco use and emphasize its negative consequences in relatable and impactful ways can be particularly effective.

Subjective norms, which reflect perceived social pressure, were positively linked to tobacco use. Students who experienced more social pressure from their family or friends to use tobacco were more likely to be current users. This is consistent with a study that observed that adolescents whose parents were highly involved in smoking cessation were 0.4 times less likely to smoke^31^. This highlights the importance of fostering a supportive environment that encourages quitting and reduces the social acceptability of tobacco use. Family and community involvement in antismoking campaigns can help alter these norms and support young people in making healthier choices^33^.

Perceived behavioral control, which reflects the ease of quitting or avoiding tobacco, was also significantly associated with tobacco use^31,34^. Students who believed they had more control over their ability to avoid tobacco were less likely to use it. However, this study observed a high perceived control among older male youths and current smokers, which is in contrast with studies that found a link between PBC and reduced smoking^32,35^. This discrepancy may be explained by the participants’ overconfidence in their ability to quit or resist tobacco use despite their actual behaviors. It may also reflect their lack of understanding of tobacco’s addictive nature, leading to a false sense of control. This suggests that programs enhancing or redirecting self-efficacy and providing coping strategies for resisting peer pressure and tobacco use can be beneficial. Additionally, educating young people on the addictive properties of tobacco and the challenges of quitting might help align their perceived control with reality, hence reducing their tobacco use.

Behavioral intentions were also predictive of actual use, indicating that students who plan to use tobacco are more likely to follow through, which is similar to a study that found that greater intention led to greater tobacco use^36^. This reinforces the need for early interventions that alter intentions before they translate into behavior. Programs that help young people set personal goals regarding tobacco use and commit to them can prevent future initiation and support current users in quitting.

### Strengths and limitations

This study has several strengths. First, its large sample of 5610 students enhances the generalizability of findings across the intermediate school population in Saudi Arabia. Second, its high response rate of 92.3% ensures that its results are likely representative of the target population. Its application of the TPB framework also provides a comprehensive understanding of the psychosocial factors that drive tobacco use among young people, offering valuable insights for public health interventions. Additionally, this study’s use of a validated and reliable instrument (GYTS) ensures data accuracy and consistency.

However, several limitations must be considered. This study’s cross-sectional design limits the ability to infer causality between TPB constructs and tobacco use; therefore, longitudinal studies must be conducted to establish causal relations. This study’s reliance on self-reported data may expose it to reporting bias as students might underreport their tobacco use because of social desirability or fear of consequences. Also, because this study was conducted only in intermediate schools and excluded other age groups and educational stages, the generalizability of the findings may be limited.

Furthermore, residual confounding remains a concern, as unmeasured variables could influence the observed associations. The use of principal component analysis (PCA) and confirmatory factor analysis (CFA), while providing robust construct validity with acceptable loadings (0.602–0.941) and average variance extracted (AVE) values >0.5, assumes linearity and may not fully account for the complexity of real-world behaviors. Lastly, the findings’ applicability to other countries is constrained by cultural and societal differences that influence tobacco use behaviors, necessitating replication of this analysis in diverse populations for broader validation.

## CONCLUSIONS

The high prevalence of tobacco uses among Saudi Arabian youth, along with early initiation and strong social influences, necessitates comprehensive and multifaceted public health strategies. These should include targeted education, policies that reduce accessibility, and programs that shift social norms and attitudes toward tobacco. By addressing the various factors identified in this study, we can better equip young people with the ability to resist the pressure to start using tobacco and support those who wish to quit.

## Supplementary Material



## Data Availability

The data supporting this research are available from the authors on reasonable request.

## References

[1] World Health Organization. Tobacco. July 31, 2023. Accessed December 1, 2024. https://www.who.int/news-room/fact-sheets/detail/tobacco

[2] Alasqah I, Mahmud I, East L, Usher K. A systematic review of the prevalence and risk factors of smoking among Saudi adolescents. Saudi Med J. 2019;40(9):867-878. doi:10.15537/smj.2019.9.24477 31522213 PMC6790477

[3] World Health Organization. WHO Report on the Global Tobacco Epidemic, 2017: Monitoring tobacco use and prevention policies. World Health Organization; 2017. Accessed December 1, 2024. https://iris.who.int/bitstream/handle/10665/255874/9789241512824-eng.pdf?sequence=1

[4] So ES, Yeo JY. Factors associated with early smoking initiation among Korean adolescents. Asian Nurs Res (Korean Soc Nurs Sci). 2015;9(2):115-119. doi:10.1016/j.anr.2015.05.002 26160239

[5] Park SH. Smoking and adolescent health. Korean J Pediatr. 2011;54(10):401. doi:10.3345/kjp.2011.54.10.401 22232621 PMC3250592

[6] Moor I, Rathmann K, Lenzi M, et al. Socioeconomic inequalities in adolescent smoking across 35 countries: a multilevel analysis of the role of family, school and peers. Eur J Public Health. 2015;25(3):457-463. doi:10.1093/eurpub/cku244 25713016

[7] Alrabah M, Gamaleddin I, Allohidan F. International approaches to tobacco-use cessation programs and policy for adolescents and young adults in Saudi Arabia. Current Addiction Reports. 2018;5:65-71. doi:10.1007/s40429-018-0188-9

[8] AlBedah AM, Khalil MK. The economic costs of tobacco consumption in the Kingdom of Saudi Arabia. Tob Control. 2014;23(5):434-436. doi:10.1136/tobaccocontrol-2012-050665 23696558

[9] Algabbani AM, Almubark RA, Althumiri NA, Alqahtani AS, BinDhim NF. The prevalence of cigarette smoking in Saudi Arabia in 2018. Food and Drug Regulatory Science Journal. 2018;1:1-13. doi:10.32868/rsj.v1i1.22

[10] Moradi-Lakeh M, El Bcheraoui C, Tuffaha M, et al. Tobacco consumption in the Kingdom of Saudi Arabia, 2013: findings from a national survey. BMC Public Health. 2015;15:1-10. doi:10.1186/s12889-015-1902-3 26141062 PMC4491232

[11] Kingdom of Saudi Arabia. Vision 2030. Health Sector Transformation Program. Accessed December 1, 2024. https://www.vision2030.gov.sa/en/explore/programs/health-sector-transformation-program

[12] Awan KH, Hussain QA, Khan S, et al. Accomplishments and challenges in tobacco control endeavors - Report from the Gulf Cooperation Council countries. Saudi Dent J. 2018;30(1):13-18. doi:10.1016/j.sdentj.2017.08.003 30166866 PMC6112370

[13] Ajzen I. The theory of planned behavior. Organizational Behavior and Human Decision Processes. 1991;50(2):179-211. doi:10.1016/0749-5978(91)90020-T

[14] Asare M. Using the theory of planned behavior to determine the condom use behavior among college students. Am J Health Stud. 2015;30(1):43. doi:10.47779/ajhs.2015.168 26512197 PMC4621079

[15] Mallery P, George D. SPSS for Windows Step by Step. Allyn & Bacon, Inc.; 2000.

[16] Nunnally JC. Assessment of reliability. In: Psychometric Theory. McGraw-Hill; 1967:206-235.

[17] Fornell C, Larcker DF. Evaluating structural equation models with unobservable variables and measurement error. Journal of Marketing Research. 1981;18(1):39-50. doi:10.1177/002224378101800104

[18] U.S. Centers for Disease Control and Prevention. Youth and Tobacco Use. Accessed December 1, 2024. https://www.cdc.gov/tobacco/php/data-statistics/youth-data-tobacco/index.html

[19] Alnasser AHA, Al-Tawfiq JA, Kheimi RMA, et al. Gender differences in smoking attitude among Saudi medical students. Asian Pac J Cancer Prev. 2022;23(6):2089-2093. doi:10.31557/APJCP.2022.23.6.2089 35763652 PMC9587824

[20] Ansari K, Farooqi FA. Comparison and prevalence of smoking among Saudi females from different Departments of the College of Applied Medical Sciences in Dammam. Int J Health Sci (Qassim). 2017;11(5):56-62. Accessed December 1, 2024. https://pmc.ncbi.nlm.nih.gov/articles/PMC5669512/pdf/IJHS-11-56.pdf 29114195 PMC5669512

[21] Al-Otaibi AA, Bin Ibrahim F, Rampal L, Hassan SA, Ibrahim N. Prevalence of tobacco use and its socio-demographic determinants among Saudi female school adolescents in Jeddah. Malaysian Journal of Medicine & Health Sciences. 2015;11(1). Accessed December 1, 2024. http://psasir.upm.edu.my/id/eprint/41445/1/Prevalence%20of%20tobacco%20use%20and%20its%20socio-demographic%20determinants%20among%20Saudi%20female%20school%20adolescents%20in%20Jeddah.pdf

[22] Leshargie CT, Alebel A, Kibret GD, et al. The impact of peer pressure on cigarette smoking among high school and university students in Ethiopia: a systemic review and meta-analysis. PLoS One. 2019;14(10):e0222572. doi:10.1371/journal.pone.0222572 31603930 PMC6788683

[23] Busch SH, Jofre-Bonet M, Falba TA, Sindelar JL. Burning a hole in the budget: tobacco spending and its crowd-out of other goods. Appl Health Econ Health Policy. 2004;3(4):263-272. doi:10.2165/00148365-200403040-00009 15901200

[24] Do YK, Bautista MA. Tobacco use and household expenditures on food, education, and healthcare in low- and middle-income countries: a multilevel analysis. BMC Public Health. 2015;15:1-11. doi:10.1186/s12889-015-2423-9 26521133 PMC4628343

[25] Xu X, Bishop EE, Kennedy SM, Simpson SA, Pechacek TF. Annual healthcare spending attributable to cigarette smoking: an update. Am J Prev Med. 2015;48(3):326-333. doi:10.1016/j.amepre.2014.10.012 25498551 PMC4603661

[26] Geboers C, Candel MJJM, Nagelhout GE, et al. Smokers’ strategies to reduce tobacco spending: self-reported use and differences across subgroups. Findings from the International Tobacco Control (ITC) Netherlands Survey. BMC Public Health. 2023;23(1):738. doi:10.1186/s12889-023-15678-9 37085828 PMC10119824

[27] World Health Organization. Raising taxes on tobacco. Accessed December 1, 2024. https://www.who.int/activities/raising-taxes-on-tobacco

[28] World Health Organization. WHO report on the global tobacco epidemic, 2021. World Health Organization; 2021. Accessed December 1, 2024. https://cdn.who.int/media/docs/default-source/country-profiles/tobacco/who_rgte_2021_saudi_arabia.pdf

[29] Niu L, Luo D, Silenzio VM, Xiao S, Tian Y. Are informing knowledge and supportive attitude enough for tobacco control? A latent class analysis of cigarette smoking patterns among medical teachers in China. Int J Environ Res Public Health. 2015;12(10):12030-12042. doi:10.3390/ijerph121012030 26404331 PMC4626953

[30] Chido-Amajuoyi OG, Osaghae I, Agaku IT, Chen B, Mantey DS. Exposure to school-based tobacco prevention interventions in low-income and middle-income countries and its association with psychosocial predictors of smoking among adolescents: a pooled cross-sectional analysis of Global Youth Tobacco Survey data from 38 countries. BMJ Open. 2024;14(2):e070749. doi:10.1136/bmjopen-2022-070749 PMC10900417 38413149

[31] Tapera R, Mbongwe B, Mhaka-Mutepfa M, Lord A, Phaladze NA, Zetola NM. The theory of planned behavior as a behavior change model for tobacco control strategies among adolescents in Botswana. PLoS One. 2020;15(6):e0233462. doi:10.1371/journal.pone.0233462 32502211 PMC7274417

[32] Riyadi S, Murti B, Akhyar M, Suminah S. Predicting tobacco smoking among adolescents using social capital and media exposure with theory of planned behavior. Global Journal of Health Science. 2019;11(7):18-28. doi:10.5539/gjhs.v11n7p18

[33] Suteerangkul P, Lagampan S, Kalampakorn S, Auemaneekul N. The effects of community participation program on smoke-free homes in a suburban community of Thailand. Tob Induc Dis. 2021;19(May):35. doi:10.18332/tid/133876 34007259 PMC8106388

[34] Hiemstra M, Otten R, de Leeuw RN, van Schayck OC, Engels RC. The changing role of self-efficacy in adolescent smoking initiation. J Adolesc Health. 2011;48(6):597-603. doi:10.1016/j.jadohealth.2010.09.011 21575820

[35] Alanazi NH, Lee JW, Dos Santos H, Job JS, Bahjri K. The use of planned behavior theory in predicting cigarette smoking among Waterpipe smokers. Tob Induc Dis. 2017;15(July):18. doi:10.1186/s12971-017-0133-z 28690480 PMC5496426

[36] Ninkron P, Yau S, Noosorn N. Predictors of smoking initiation among Thai adolescents from low-income backgrounds: a case study of Nakhon Pathom low-cost housing estates. Tob Induc Dis. 2022;20(February):21. doi:10.18332/tid/145143 35280045 PMC8861864

