# Supplementary material for: Factors associated with tobacco use among Saudi Arabian youth: Application of the Theory of Planned Behavior
Source: Tob Induc Dis. 2024 Dec 18;22:10.18332/tid/196678. doi: 10.18332/tid/196678 (PMC11653066; doi:10.18332/tid/196678)
Supplement: Supplementary file 1 [file TID-22-189-s1.pdf]

Supplementary file

In this annex, detailed information is provided about each construct in the questionnaire. For each construct, the number of items included and the Cronbach's alpha are specified, indicating the scale's reliability and the content covered by each construct is described.

Additionally, the factor loadings and maximum scores are presented for each construct. The factor loadings indicate the strength of the relationship between each item and its corresponding construct. The maximum score represents the highest possible score that participants can achieve, reflecting the range of responses they can provide.

Measurement and Reliability Assessment of TPB constructs

	No of items	Score (Min.-Max.)	Cronbach's alpha
**Behavioural Intentions**	3	3-12	0.731
**Attitude**	7	7-27	0.695
**Subjective Norms**	9	9-33	0.795
**Perceived Behavioural Control**	2	2-8	0.566
**Awareness and Knowledge**	16	0-16	0.570

1. Intentions

	No of items	Cronbach's alpha
**Behavioural Intentions**	3	0.731

**Behavioural Intentions**	**Max. score**	**Loading**
During the next 12 months, do you think you will use any form of tobacco?	4	0.692
In the past 30 days, have you tried to quit smoking?	4	0.869
Do you want to stop smoking now?	4	0.868

2. Attitude

	No of items	Cronbach's alpha
**Attitude about enjoyment and attraction**	3	0.527
**Attitude about quitting difficulty**	4	0.935
**Overall**	7	0.695

	Max. score	Loading
**Enjoy- attraction**		
Do you agree or disagree with the statement: "I think I might enjoy smoking a cigarette	5	0.749
Do you think smoking tobacco makes people feel more or less comfortable at parties and social events?	3	0.713
Do you think smoking tobacco makes young people look more or less attractive	3	0.697
	**11**	
**Quitting**		
Once someone starts **smoking**, do you believe it will be difficult for them to stop	4	0.890
Once someone starts smoking **shisha**, do you believe it will be difficult for them to stop smoking it?	4	0.941
Once someone starts using **electronic cigarettes**, do you believe it will be difficult for them to stop using them?	4	0.927
Once someone starts using **non-smoked tobacco**, do you believe it will be difficult for them to stop	4	0.900
	**16**	

3. Subjective Norms

	No of items	Cronbach's alpha
**Family-peers effect**	2	0.343
**Best friend offers**	4	0.843
**Social offers**	3	0.730
**Overall**	9	0.795

	Max. score	Loading
**Family-peers effect**		
Do any of your best friend's smoke cigarettes	4	0.778
Do your parents or guardians smoke cigarettes?	4	0.776
**Best friend offers**		
If one of your close friends gave you a tobacco product, would you use it?	4	0.806
If a close friend offered you to smoke shisha, would you do it?	4	0.814
If a close friend offered you electronic cigarettes, would you use them?	4	0.805
If a close friend offered you non-smoked tobacco, would you use it?	4	0.733
**Retailer salesmen effect**		
In the past 30 days, has anyone refused to sell you cigarettes because of your age	**3**	0.816
Has anyone refused to offer you shisha because of your age?	**3**	0.792
Has anyone refused to sell e-cigarettes due to age?	**3**	0.740

4. Perceived Behavioural Control

	No of items	Cronbach's alpha
**Perceived Behavioural Control**	2	0.566

	Max. score	Loading
**Perceived Behavioural Control**		
Do you ever smoke tobacco or feel like smoking tobacco first thing in the morning?	4	0.616
Do you think you can quit smoking if you want?	4	0.874

5. Awareness and Knowledge

	No of items	Cronbach's alpha
**Knowledge about health hazards of smoking**	4	0.659
**Awareness of messages that are against using tobacco**	5	0.570
**Awareness of advertisements or promotions for tobacco**	7	0.500

**Knowledge about health hazards of smoking**	**Loading**
Do you think that exposure to smoke from others smoking is harmful to your health?	0.785
Do you think that exposure to shisha (hookah) smoke is harmful to your health?	0.798
Are you in favor of banning smoking inside enclosed public places (Such as shops, restaurants, shopping malls, cinemas, theaters, cafes, rest houses - government buildings)?	0.674
Are you in Favor of banning smoking at outdoor public places (such as parks, building entrances, resorts, beaches)?	0.610
**Awareness of messages that are against using tobacco**	
During the past 30 days, did you see or hear any anti-tobacco media messages on television, radio, internet, social media, billboards, posters, newspapers, magazines, or movies?	0.602
During the past 30 days, did you see or hear any anti-tobacco messages at sports events, fairs, concerts, community events, or social gatherings?	0.612
During the past 30 days, did you see any health warnings on cigarette packages?	0.797
During the past 30 days, did you see any health warnings on shisha tobacco packages?	0.780
During the past 12 months, were you taught in any of your classes about the dangers of tobacco use?	0.616
**Awareness of advertisements or promotions for tobacco**	
During the past 30 days, did you see any people using tobacco when you watched TV, videos, or movies?	0.782
During the past 30 days, did you see any advertisements or promotions for tobacco products at points of sale (such as Stores, shops, websites)?	0.735
Would you ever use or wear something that has a tobacco company or tobacco product name or picture on it such as a lighter, t-shirt, hat, or sunglasses?	0.635
Do you have something (for example, t-shirt, pen, backpack) with a tobacco product brand logo on it?	0.646

Has a person working for a tobacco company ever offered you a free tobacco product?	0.828
Has a person working for a tobacco company ever offered you a free e-cigarette product?	0.836
Has a person working for a tobacco company ever offered you a free heated tobacco product?	0.793

© 2024 Shubayr M.A. et al.
